# Six Months of Hospital War Work

**Published:** 1915-03-20

**Authors:** 


### SIX MONTHS OF HOSPITAL WAR WORK.

Now that we know that a large additional number of beds for the wounded is being arranged for all over the country, it is perhaps well to cast a glance over the relations which have existed between the voluntary hospitals and the military authorities during the last six months. For it would seem that the first phase of hospital preparation is coming to an end, and that a second phase on a larger scale, and in the light of acquired experience, is beginning. From the point of view of the voluntary hospitals—at any rate in London—it cannot be denied that there has been a degree of disappointment, and the general reasons to which this disappointment is due are worth recording.

At the outbreak of war the hospitals were approached, and offered to do all in their power to assist the military authorities. They made, as a rule, a free offer of their accommodation and services, and the result was that a little later, when ways and means began to assume serious proportions, their overtures for payment were met by the reply that the War Office would give preference in the matter of patients to those institutions to which no payment was being made. This attitude was naturally somewhat resented. Why, it was asked, should the War Office place upon charitable institutions very great additional burdens without contributing to the cost entailed by them?

The number of patients which it became necessary to treat, however, did, as we all know, soon fill many hospital wards. But what was the position of the hospitals in respect of these cases? There was, on the one hand, a series of institutions in which the best available skill and the most complete equipment were to be found; on the other was the decision on the part of the authorities not, as a whole, to entrust to their care any but minor cases. There may have been reasons for this; no doubt there were; but the hospital staffs, we submit, had and still have some reason to regret that the magnificent resources which they have placed at the disposal of our wounded should, in the main, have been passed over by confining the patients admitted to their wards so largely to cases of frost-bite and minor ailments.

Then, again, matters have not been made easy for the voluntary hospitals in the arrangements imposed on them for dealing with the wounded. An elaborate system of forms has been devised, and much correspondence has been necessitated, so that the arrangements which have been permitted to the London Hospital, recorded on p. 555, come as an exceptional and welcome example of the simplification of procedure. For it cannot be doubted that such simplification is all to the good of the military and hospitals alike, and now that the London hospitals are beginning to press their claims for payment with some prospect of success, it is to be hoped that the procedure may be simplified all round. While much money has been spent on adapting various buildings all over the country, the hospitals have not received very generous treatment, and the only consolation to be drawn from this fact is the indirect reminder thus provided that, after all, their primary duty must be to the civilian population. The above sketch of the position, as it has presented itself to many hospitals since their offers to receive the wounded were accepted, provides an outline of what has been happening during the past six months in this direction. Is it too much to hope that the future and probably extended arrangements may be concluded in a wider spirit and conducted on more simplified and, consequently, more efficient lines?

